# 

**Published:** 1863-11

**Authors:** 


### BOOKS AND PAMPHLETS RECEIVED.

*The Principles and Practice of Ophthalmic Medicine and Surgery.* By T. Wharton Jones, F. R. S., *Professor of Ophthalmic Medicine and Surgery in University College, London ; Ophthalmic Surgeon to the Hospital, etc., with One Hundred and Seventeen Illustrations. Third and Revised American Edition, with additions from the Second London Edition. Philadelphia:* Blanchard & Lea, 1863.

*Mental Hygiene.* By J. Ray, M. D. *Boston:* Ticknor & Fields, 1863.

*A Case of Neuroma of the Optic Nerve, with remarks and Illustrations.* By John A. Lidell, M.D., *Surg. U. S. Vol., Prof. of Anatomy in the National Medical College. Second Edition.*

*Illustrated Catalogue of Medical, Surgical, and Scientific Publications. Philadelphia:* Blanchard & Lea, 1863.

*The Union Monthly, and Journal of Health and Education, devoted to the Union of the Nation, National Education, and the Temporal and Spiritual Welfare of the Army.* Wm. M. Cornell, M.D., LL.D. Editor. *Philadelphia and Boston,* 1863.

*Annual Announcement of the Medical Department of the University of the Pacific, San Francisco, Calafornia. Session of* 1863–64. *San Francisco:* Towne & Bacon, 1863.

*The Sanitary Commisson Bulletin.*

*Peterson's Ladies National Magazine. For December.*

*Godey's Ladies Book. For December.*

*Atlantic Monthly. For December.*

